# No evidence of a clinically important effect of adding local infusion analgesia administrated through a catheter in pain treatment after total hip arthroplasty: A randomized double-blind and placebo-controlled clinical trial involving 60 patients

**DOI:** 10.3109/17453674.2011.570671

**Published:** 2011-07-08

**Authors:** Kirsten Specht, Jane Schwartz Leonhardt, Peter Revald, Hans Mandøe, Else Bay Andresen, John Brodersen, Svend Kreiner, Per Kjaersgaard-Andersen

**Affiliations:** ^1^Clinical Research Unit, Department of Orthopaedics; ^2^Department of Anesthesiology, Vejle Hospital; ^3^Department and Research Unit of General Practice; ^4^Department of Biostatistics, Institute of Public Health, University of Copenhagen, Denmark

## Abstract

**Background and purpose:**

Postoperative analgesia after primary total hip arthroplasty (THA) using opioids is associated with troublesome side effects such as nausea and dizziness, and epidural analgesic means delayed mobilization. Thus, local infiltration analgesia (LIA) during surgery prolonged with local infusion analgesia (LINFA) into the soft tissue in the hip region through a catheter in the first postoperative days has gained major interest in THA fast-track settings within a short period of time. LIA at the time of surgery is a validated treatment. We investigated the additional effect of giving postoperative LINFA after THA in patients already having LIA during surgery.

**Patients and methods:**

60 consecutive patients undergoing non-cemented THA were randomized into two groups in a double-blind and controlled study. During surgery, all patients received standardized pain treatment with LIA. Postoperatively, they were treated either with a solution of Ropivacain, Ketorolac, and Adrenaline (LINFA group) or placebo (placebo group) administered through a catheter to the hip 10 and 22 h after surgery. Pain score, opioid consumption, and length of stay (LOS) were evaluated.

**Results:**

After adjustment for multiple testing, there was no statistically significant postoperative difference between the LINFA group and the placebo group regarding pain and tiredness. We found some evidence of a short-term effect on nausea and vomiting. Opioid consumption and length of stay were similar in the two groups.

**Interpretation:**

We found some evidence of a short-term effect of LINFA on nausea and vomiting, but no evidence of an effect on postoperative pain and tiredness. Thus, LINFA cannot be recommended as a standard pain treatment in patients with THA.

In Denmark, the waiting time for total hip arthroplasty (THA) early in the last decade was more than 1 year. To increase the numbers of surgeries, several hospitals focused on early mobilization and early discharge. Because of this, perioperative pain treatment gained major attention as the most important factor (Kehlet and Dahl 2003, Rostlund and Kehlet 2007).

Postoperative pain in THA has traditionally been managed with epidural analgesia (Choi et al. 2003), with peripheral nerve blocks, or solely with opioid drugs (Fischer and Simanski 2005). Opioids are well known to be associated with troublesome side effects such as nausea, vomiting, and dizziness, and epidural analgesic is associated with urinary retention and delayed mobilization. Thus, neither opioids nor epidural pain treatment are attractive as a treatment modality in units focusing on fast rehabilitation after THA. The next generation of analgesia regimes in THA, with reduced or minimal side effects while maintaining adequate pain relief and maximum muscle control, is therefore of considerable interest.

Local infiltration analgesia (LIA) was presented by Kerr and Kohan of the Joint Orthopaedic Center in Sydney, Australia, in 2005 (Kerr and Kohan 2008). They reported a multimodal technique including LIA as pain relief in a fast-track setting after total hip and knee replacement. In their intraoperative set-up, soft tissues around the surgical field were infiltrated with a mixture of Ropivacaine, Ketorolac, and adrenaline, followed by LINFA through a catheter after 20 h. In a non-randomized study, they reported effective pain relief with early mobilization and reduced length of stay (LOS) (Kerr and Kohan 2008).

Three other publications have presented pain results after LIA in THA (Bianconi et al. 2003, Andersen et al. 2007a, b). In 2003, in a randomized study with a limited number of patients, Bianconi et al. showed intraoperative LIA in combination with postoperative LINFA with Ropivacaine to be superior to intravenous infusion of morphine and Ketorolac postoperatively (Bianconi et al. 2003). In a randomized, double-blind study, Andersen et al. 2007a) showed intraoperative infiltration with a mixture of Ropivacaine, Ketorolac, and adrenaline into the deep tissue in the wound followed after 8 h by intraarticular LINFA with the same mixture through a catheter to be more effective than continuous epidural infusion with Ropivacaine and morphine. Finally, in a randomized, double-blind study Andersen et al. (2007b) found intraoperative LIA with a mixture of Ropivacaine, Ketorolac, and adrenaline combined with postoperative LINFA using same mixture through a catheter in the hip joint to be substantially more effective than saline administered both intraoperatively and postoperatively.

Within a short period, LIA during surgery combined with LINFA into the soft tissue in the hip region through a catheter in the first postoperative days has gained major interest in fast-track THA settings. However, there is no evidence from the existing literature of there being any extra benefit from adding postoperative LINFA in patients who have already been treated with LIA during surgery.

We investigated whether there was any additional effect of LINFA administered postoperatively through a catheter on pain and opioid consumption over the first 24 h, and its effect on length of hospital stay after THA in patients already treated with LIA during surgery.

## Patients and methods

The study protocol was approved by the Ethics Review Board of Southern Denmark (June 22, 2007, ID S-20070066) and the Danish Drug Agency (EudraCT no. 2007-003890-20). It was registered with ClinicalTrials.gov (ID NCT00603083) and conducted in accordance with the Declaration of Helsinki. After written informed consent was obtained, 60 consecutive patients over 18 years of age with osteoarthritis of the hip, who were scheduled for uncemented unilateral THA, were enrolled. Primary exclusion criteria were: anterior surgical approach, use of navigation equipment during surgery, known allergy or intolerance to one of the study drugs, simultaneous bilateral THA, general anesthesia, regular use of opioids, inability to comprehend pain scales, women of fertile age, an American Society of Anesthesiologists (ASA) classification of III or more, medication with lithium, dihydroergotamine or anticoagulants, active peptic ulcer, hemorrhagic diastase, gastrointestinal bleeding, severe thrombocytopenia, bleeding disorders, asthma, severe liver or heart disease, hypertension, or thyrotoxicosis.

Surgery was performed at the Department of Orthopaedic Surgery, Vejle Hospital, from January, 2008 through October, 2008, and for this study patients were followed for 7 days after surgery.

### Randomization and blinding

The study design was a randomized, double-blind and placebo-controlled clinical trial. The randomization was carried out by an external dispensary using a computer-generated second-generation randomization with 10 blocks of 6 patients each (www.randomization.com). The allocation outcome was concealed from the patients, the surgeons, and all healthcare providers, data collectors, assessors of outcome, and statisticians (Haahr and Hrobjartsson 2006). On the day of surgery, the patients were randomized to receive either LINFA or placebo by using sequentially numbered medicine bottles produced and labeled by the external dispensary. The allocation outcome was concealed until all results were observed, recorded, and analyzed. During this period, the groups were named A and B.

### Anesthesia

All patients had their hips operated using a combined spinal anesthesia (1.5 mL Bupicaine 0.25% + 1.5 mL saline at L2/3 or L3/4, 25 gauge, Quincke tip) and light general anesthesia (a combination of Propofol and Ketamine infusion). If there was airway obstruction, a laryngeal mask airway was used.

### Surgery

All operations were performed by one of 4 surgeons using a posterolateral approach. The same surgical technique was used in both groups, including absence of suction drainage from the wounds.

### Pain treatment

On the morning of surgery, Paracetamol Retard (2 g) was given orally to both groups as premedication. During surgery, all patients in both groups received a standardized pain treatment with LIA (200 mg Ropivacaine (2 mg/mL), 30 mg Ketorolac (30 mg/mL), and 1 mg adrenaline (1mg/mL)) as a total solution of 102 mL, which was divided into in 2 injections. After implanting the acetabular component, the first 51 mL of LIA was injected; the second injection of 51 mL was given after reinsertion of the rotator muscles. Standardized techniques for the injection were used, with the first dose being given mainly in the anterior and inferior joint capsule, and the lesser gluteal muscle. The second dose was administered to the medius and maximus gluteal muscle and the lesser rotators. All patients had a tunneled intraarticular multi-hole catheter placed by the surgeon at the end of surgery. As postoperative oral pain treatment, all patients received 1 g Paracetamol 4 times a day, starting in the post-anesthesia care unit, and Ibuprofen 400 mg 4 times a day, starting in the morning after the operation. Oxycodone (5 mg) was used as patient-controlled rescue medication. After 10 and 22 h, 51 mL of the same solution as used during surgery was injected intraarticularly through the catheter in the LINFA group. In the placebo group, a similar volume of saline was injected at that same time. All patients had the catheter removed after the last injection was given.

### Outcome measures

*Opioid consumption* was registered by the patients themselves followed by a check by the nurse on duty. Consumption was recorded during the first 72 h after surgery. For an overall evaluation, narcotics were converted to morphine equivalents (Schug and Gandham 2006).

*Pain* was evaluated by using a self-reported questionnaire: Western Ontario MacMaster Universities (WOMAC verbal rating scale) osteoarthritis index (Bellamy et al. 1988) score for pain preoperatively, as primary endpoints at 12, 22, and 24 h postoperatively, and after that as secondary endpoints at 8 a.m. and 8 p.m. until day 3 after surgery. The last endpoint for pain scores was 7 days after surgery. In addition, until day 7 at those same time points, the numerical rating scale (NRS 0–10) was used for pain score at rest and during activity (first 12 h after surgery: trying to raise leg from the mattress; after 12 h: walking).

*Postoperative nausea and vomiting (PONV).* NRS (0–10) and 3 items from the European Organization for Research and Treatment of Cancer (EORTC) QLQ-C30 (Sprangers et al. 1993) were used to score PONV at 8 p.m. on days 0–3 after surgery.

*Tiredness.* NRS (0–10) and 3 items from the Nottingham Health Profile (NHP) (Hunt et al. 1986, Thorsen et al. 1993) were used for assessment of tiredness preoperatively and at 8 p.m. on days 0–3 after surgery.

*LOS.* The day of surgery was defined as day 0. The patients were discharged when they had fulfilled the following criteria: walking with elbow crutches, ability to climb stairs, and no evidence of any surgical complications.

All complications and adverse events were registered intraoperatively, postoperatively and after discharge.

### Statistics

A power analysis was done before the start of the study using Oxycodone consumption as the primary endpoint. With an a of 0.05 and a β of 0.2, we expected a reduction in Oxycodone from 38 mg to 19 mg in the treatment group. This 50% reduction in opoid consumption was regarded as a relevant clinically significant effect, and was used as the primary endpoint. With a standard deviation of 23 mg, 22 patients were needed in each group. Assuming that there would be a somewhat greater standard deviation in the control group, we decided to include 30 patients in each group.

Rasch analyses (Rasch 1960) were conducted on the WOMAC pain scale and the 2 NRS pain items, on the 3 nausea items from the EORTC plus the nausea NRS, and on the 3 energy items from the NHP plus the tiredness NRS. The Rasch analyses included examination of dimensionality, local response dependencies, and differential item functioning (DIF). Fit to the Rasch model was estimated by comparing the observed and expected responses for each item evaluated by conditional likelihood ratio chi-square test. Conditional likelihood ratio chi-square test was also used for overall tests of DIF. The tests were done both for the partial credit Rasch model and for the log-linear Rasch model (Kreiner and Christensen 2007, Kreiner 2007).

For each of the 3 constructs, all items fitting the Rasch model were used to obtain the most valid and reliable measurement of pain, nausea, and tiredness. Inclusion of all Rasch-fitting items would cover the latent trait measured with most response categories from the polytomous items included in the dimensions. In this way, maximum achievable valid information of each construct was ensured.

Data were analyzed with an intention-to-treat approach. The Mann-Whitney U test was used for analysis of both the primary and secondary endpoints, since the data were not normally distributed. Results are presented as frequency (%) or median (range). Any p-values of < 0.05 were considered to be statistically significant.

## Results

### Item analysis by graphical log-linear Rasch models

*Pain measurement.* There was strong evidence of local dependence between the WOMAC items 1 and 7, 2 and 3, 4 and 6, and 5 and 6. There was little and inconsequent evidence of DIF among the WOMAC items. All WOMAC items fitted the log-linear Rasch model. The local dependence among several of the items meant that the reliability was lower than suggested by Cronbach's alfa (a = 0.94, true reliability = 0.90). The two NRS pain items measured the same latent trait as the WOMAC pain scale (expected pain score = 0.730, observed pain score = 0.738, p = 0.6 by chi-squared test) with no further local response dependency and DIF revealed. All WOMAC items plus the two NRS items therefore appeared to be preferable for measurement of pain.

*Nausea measurement.* Tests of the conditional likelihood ratio comparing item parameter estimates in different subpopulations defined by total scores on the EORTC nausea scale, by different time points and by values of exogenous covariates, revealed evidence against the Rasch models (c^2^ = 17.7, df = 8, p = 0.02). No DIF among the 3 EORTC items was revealed; however, items 1 and 2 were locally dependent. The log-linear Rasch model perfectly predicted the strength of the association between EORTC items and rest scores (Table 1, see supplementary data). Adding the NRS nausea item to the scale did not change anything: no DIF, but local dependence between the first 2 items. The reliability of the 3-item scale was 0.81. The reliability of the global item was 0.88. All EORTC items plus the NRS items therefore appeared to be preferable for measurement of nausea.

*Tiredness measurement.* No evidence of local dependency among the 3 NHP energy items was revealed and there was no evidence against the Rasch models. However, comparison of separate NHP items over time and across age groups disclosed strong evidence of DIF of item 1 relative to age (p = 0.001) and of item 3 relative to time of measurement (p < 0.001). The DIF relative to time is particularly disturbing, since it will confound measurement of the general energy levels over time if this is disregarded.

The fit of the 3 NHP items and the NRS tiredness item to the partial credit model was acceptable, except that the DIF for the 2 above-mentioned NHP items reappeared. Assessment of reliability of the 3-NHP-item scale was r = 0.76 while the reliability of the global question was r = 0.94. For these two reasons, the global question appeared to be preferable for measurement of tiredness.

### Patients

Of the 60 patients enrolled in the study, 4 in the placebo group and 2 in the LINFA group discontinued the intervention (Figure, see supplementary data). In the placebo group, 2 patients did not receive the second postoperative intraarticular injection, one by mistake and the other because the patient did not want it. 2 other patients in the placebo group did not receive any of the 2 intraarticular injections due to a blocked catheter. In the LINFA group, 2 patients did not receive either of the 2 postoperative intraarticular injections: 1 had a blocked catheter and the other had general anesthesia and did not get a catheter. Data from all 60 patients were analyzed (Figure, see supplementary data).

The placebo group had more females (60%) than the LINFA group (33%), and the patients in the placebo group were older with less function than the patients in the LINFA group (Table 2). The 2 groups were similar regarding body mass index, living with a partner, ASA classification, preoperative pain level, duration of surgery, and intraoperative bleeding (Table 2).

**Table 2.** Patient characteristics for the LINFA and placebo group

	LINFA group	Placebo group
	(n = 30)	(n = 30)
Age, years **^a^**	64 (54–78)	68 (54–82)
Female, n (%)	10 (33%)	18 (60%)
Body mass index **^a^**	27 (19–37)	28 (20–37)
Living with partner, n (%)	24 (80%)	25 (83%)
ASA classification, n (%)		
I (normal healthy)	8 (27%)	11 (37%)
II (mild systemic disease)	22 (73%)	19 (63%)
Preoperative pain level **^a^**		
WOMAC	13.0 (1–20)	13.0 (4–18)
NRS (rest + activity)	7.5 (0–16)	9.0 (0–15)
WOMAC + NRS (rest + activity)	20.5 (2–34)	22.0 (6–31)
Preoperative WOMAC **^a^**		
Stiffness	8 (0–12)	8 (0–14)
Function	33 (8–58)	40 (3–60)
Duration of surgery, min **^a^**	60 (35–110)	50 (30–90)
Intraoperative bleeding, mL **^a^**	250 (50–750)	200 (50–1,100)

**^a^** Median (range).

### Consumption of opioids

During the first 24 h after surgery, no statistically significant difference in opioid consumption was observed between the 2 groups (p = 0.5). In all other measured periods until day 7, no significant difference was observed (Table 3, see supplementary data).

### Postoperative pain

After adjustment for multiple testing, there was no statistically significant difference between the LINFA group and the placebo group regarding postoperative pain (Tables 4 and 5, see supplementary data).

**Table 4.** Results for postoperative pain as primary endpoint, median (range)

Pain scale	LINFA group	Placebo group	n (L/P) **^a^**	p-value **^b^**
12 hours				
WOMAC	3.5 (0–7)	2.5 (0–5)	28/28	0.6
22 hours				
WOMAC	7 (2–15)	9 (0–13)	24/24	0.2
NRS **^c^**	5 (1–16)	8 (0–18)	23/27	0.1
WOMAC + NRS **^c^**	12 (3–30)	17 (0–31)	23/23	0.1
24 hours				
WOMAC	4 (0–12)	6 (0–10)	29/28	0.05 **^d^**
NRS **^c^**	4 (0–14)	6 (0–17)	30/27	0.04 **^d^**
WOMAC + NRS **^c^**	9 (0–23)	12.5 (0–26)	29/26	0.05 **^d^**

**^a^** n: number of registrations in the two groups (LINFA/placebo).

**^b^** Mann-Whitney U test.

**^c^** NRS pain (rest + activity)

**^d^** Adjustment of the p-values in the table in order to control the false discovery rate and so avoid spurious significant results due to multiple testing suggested that these results should be regarded as insignificant (Benjamini and Hochberg 1995).

### Adverse effects

On days 1 and 2 after surgery, there were higher incidences of nausea and vomiting in the placebo group than in the LINFA group (Table 6). After adjustment for multiple testing, there was no statistically significant difference between the LINFA group and the placebo group regarding tiredness postoperatively (Table 6).

**Table 6.** Results for PONV and tiredness in the LINFA and placebo groups, median (range)

	LINFA group	Placebo group	n (L/P) **^a^**	p-value **^b^**				
Nausea + vomiting (EORTC)				
Day 0	1 (0–9)	2 (0–9)	30/30	0.1
Day 1	0 (0–6)	2 (0–9)	30/30	< 0.001 **^c^**
Day 2	0 (0–3)	1 (0–5)	30/30	0.001 **^c^**
Day 3	0 (0–8)	0 (0–4)	28/29	0.5
Nausea (NRS)				
Day 0	0.5 (0–8)	1.5 (0–10)	30/30	0.2
Day 1	0 (0–8)	2 (0–9)	30/30	0.002 **^c^**
Day 2	0 (0–2)	0.5 (0–6)	30/30	0.01 **^c^**
Day 3	0 (0–10)	0 (0–6)	28/29	0.6
Nausea + vomiting (EORTC + NRS)				
Day 0	1.5 (0–17)	3.5 (0–19)	30/30	0.2
Day 1	0 (0–13)	4 (0–17)	30/30	< 0.001 **^c^**
Day 2	0 (0–5)	1.5 (0–10)	30/30	0.004 **^c^**
Day 3	0 (0–18)	0 (0–9)	28/29	0.6
Tiredness (NRS)				
Day 0	3 (0–10)	4 (0–9)	29/29	0.7
Day 1	3 (0–9)	4 (0–9)	30/30	0.04 **^d^**
Day 2	2 (0–6)	3 (0–8)	30/30	0.08
Day 3	2 (0–7)	2 (0–8)	28/28	0.3

**^a^** n: Number of registrations in the two groups (LINFA/placebo).

**^b^** Mann-Whitney U test.

**^c^** Statistically significant.

**^d^** Adjustment of the p-values in the table in order to control the false discovery rate and so avoid spurious significant results due to multiple testing suggested that this result should be regarded as insignificant (Benjamini and Hochberg 1995).

None of the 60 patients had any evidence of a prosthesis infection. 1 patient in the placebo group was treated with antibiotic because of prolonged wound drainage.

### LOS

The study showed a trend of shorter LOS in the LINFA group (median 3 (2–6) days) than in the placebo group (median 3 (2–7) days) but the difference was not statistically significant (p = 0.09).

## Discussion

We found similar opioid consumption and postoperative pain scores in the LINFA group and the placebo group. Patients having LIA during surgery and also being treated with LINFA through a catheter 10 and 22 h after surgery were a priori expected to have less pain and/or reduced consumption of opioids than the placebo-treated patients. Those in both groups were given LIA during surgery. This may explain why no difference in pain was observed in the first 24 h after surgery, as LIA may still be effective at this early postoperative period.

In a randomized, double-blind study Andersen et al. (2007b) investigated the effect of pain treatment with LIA combined with LINFA. Patients were randomized to receive either LIA combined with LINFA or saline administered during surgery and 24 h after THA surgery. The study involved 40 patients. A reduced consumption of Oxycodon on days 1–4 and also reduction in pain 4 h after surgery and for up to 2 weeks after surgery was found in the group receiving LIA combined with LINFA.

In another randomized and double-blind study involving 37 patients with THA and total knee arthroplasty, Bianconi et al. 2003) investigated the effect of LIA given during surgery in combination with 55 h of continuous LINFA administered through a catheter in the hip. This pain treatment was compared with intravenous morphine treatment, and the conclusion was that the consumption of additional analgesic 0–72 h after surgery was reduced; less pain was also reported 8–72 h after surgery in patients treated with LIA in combination with LINFA.

Andersen et al. (2007a) studied 80 THA patients to investigate the effect of LIA given during surgery in combination with LINFA (administered as 1 injection through a catheter to the hip 8 h after surgery). Pain treatment using LIA combined with LINFA was compared with continuous epidural pain treatment, and there was less pain 24–96 h after surgery and reduced opioid consumption 0–96 hours after surgery in patients treated with LIA combined with LINFA.

It is not possible to compare the 3 studies above (Bianconi et al. 2003, Andersen et al. 2007a, b) directly with the present study, as their design differs. The 3 studies investigated the total effect of LIA given during surgery in combination with LINFA administered through a catheter as injections or continuous infusion after surgery. Our study, in which all patients in both groups received LIA during surgery, is the first to focus only on the effect of treatment with LINFA given after surgery. We could not show the same positive effect of postoperative LINFA on pain relief as the three other studies. This may be explained by the fact that pain treatment with LIA during surgery is highly effective, with an extended postoperative “hangover” pain-reducing effect, making postoperative treatment with LINFA (administered through a catheter into the hip) of minor or no importance.

There was a higher proportion of women in the placebo group, which may have contributed to the findings of the difference in postoperative nausea and vomiting between the 2 groups. It is well known that women are much more susceptible to PONV than men (Gan et al. 2003).

The median LOS in our study was 3 days for both groups, although the LINFA group had a slightly shorter LOS—but not statistically significantly. These LOS values are shorter than the LOS gathered on a national basis for 2004, which was mean 7.4 (4.6–10.9) days at that time (Husted et al. 2006). The reason why we can not find a statistically significant difference in LOS might be because the LOS already is very low and to reduce that even further it is required to make larger changes probably at the same time.

Our data were analyzed with an intention-to-treat approach. 60 patients were included in the analysis, but there were 6 breaches of protocol and the actual study group was 26 in the placebo group and 28 in the LINFA group. Thus, a per protocol analysis was done. The results from the per protocol analysis were similar to those from the intention-to-treat results.

163 patients were evaluated for participation in this study. Of these, 103 were immediately excluded, mainly due to preoperative use of opioids, surgery in general anesthesia, or treatment with different drugs that might possibly interfere with the study drugs. This may have weakened the final outcome of the study, but in contrast it may have strengthened the “signal” in the patients under investigation where no possible bias from other medications could have arisen.

In summary, we found some evidence of a short-term effect of LINFA on nausea and vomiting, but there was no evidence of an effect on postoperative pain and tiredness. Thus, LINFA cannot be recommended as a standard pain treatment in patients undergoing THA.

## Supplementary data

Tables 1, 3, and 5 and the Figure are available at our website (www.actaorthop.org), identification number 4132.

## References

[CIT0001] Andersen KV, Pfeiffer-Jensen M, Haraldsted V, Soballe K (2007a). Reduced hospital stay and narcotic consumption, and improved mobilization with local and intraarticular infiltration after hip arthroplasty: A randomized clinical trial of an intraarticular technique versus epidural infusion in 80 patients. Acta Orthop.

[CIT0002] Andersen LJ, Poulsen T, Krogh B, Nielsen T (2007b). Postoperative analgesia in total hip arthroplasty: A randomized double-blinded, placebo-controlled study on peroperative and postoperative ropivacaine, ketorolac, and adrenaline wound infiltration. Acta Orthop.

[CIT0003] Bellamy N, Buchanan WW, Goldsmith CH, Campbell J, Stitt LW (1988). Validation study of WOMAC: A health status instrument for measuring clinically important patient relevant outcomes to antirheumatic drug therapy in patients with osteoarthritis of the hip or knee. J Rheumatol.

[CIT0004] Benjamini Y, Hochberg Y (1995). Controlling the false discovery rate: A practical and powerful approach to multiple testing. J R Statist Soc B.

[CIT0005] Bianconi M, Ferraro L, Traina GC, Zanoli G, Antonelli T, Guberti A, Ricci R, Massari L (2003). Pharmacokinetics and efficacy of ropivacaine continuous wound instillation after joint replacement surgery. Br J Anaesth.

[CIT0006] Choi PT, Bhandari M, Scott J, Douketis J (2003). Epidural analgesia for pain relief following hip or knee replacement. Cochrane Database Syst Rev.

[CIT0007] Fischer HB, Simanski CJ (2005). A procedure-specific systematic review and consensus recommendations for analgesia after total hip replacement. Anaesthesia.

[CIT0008] Gan TJ, Meyer T, Apfel CC, Chung F, Davis PJ, Eubanks S, Kovac A, Philip BK, Sessler DI, Temo J, Tramer MR, Watcha M (2003). Department of Anesthesiology, Duke University Medical Center. Consensus guidelines for managing postoperative nausea and vomiting. Anesth Analg.

[CIT0009] Haahr MT, Hrobjartsson A (2006). Who is blinded in randomized clinical trials? A study of 200 trials and a survey of authors. Clin Trials.

[CIT0010] Hunt SM, McEwen J, McKenna SP (1986). Measuring health status.

[CIT0011] Husted H, Hansen HC, Holm G, Bach-Dal C, Rud K, Andersen KL, Kehlet H (2006). Length of stay in total hip and knee arthroplasty in danmark I: Volume, morbidity, mortality and resource utilization. A national survey in orthopaedic departments in denmark. Ugeskr Laeger.

[CIT0012] Kehlet H, Dahl JB (2003). Anaesthesia, surgery, and challenges in postoperative recovery. Lancet.

[CIT0013] Kerr DR, Kohan L (2008). Local infiltration analgesia: A technique for the control of acute postoperative pain following knee and hip surgery: A case study of 325 patients. Acta Orthop.

[CIT0014] Kreiner S (2007). Item analysis in DIGRAM; preliminary version.

[CIT0015] Kreiner S, Christensen KB, von Davier M, Carstensen CH (2007). Validity and objectivity in health-related scales: Analysis by graphical loglinear rasch models. In Multivariate and mixture distribution Rasch models: Extensions and applications.

[CIT0016] Rasch G (1960). Probabilistic models for some intelligence and attainment tests.

[CIT0017] Rostlund T, Kehlet H (2007). High-dose local infiltration analgesia after hip and knee replacement--what is it, why does it work, and what are the future challenges?. Acta Orthop.

[CIT0018] Schug SA, Gandham N (2006). Opioids: Clinical use. In: Wall and Melzack's textbook of pain (Eds McMahon SB, Koltzenburg M).

[CIT0019] Sprangers MA, Cull A, Bjordal K, Groenvold M, Aaronson NK (1993). The european organization for research and treatment of cancer. approach to quality of life assessment: Guidelines for developing questionnaire modules. EORTC study group on quality of life. Qual Life Res.

[CIT0020] Thorsen H, McKenna SP, Gottschalck L (1993). The danish version of the nottingham health profile: Its adaptation and reliability. Scand J Prim Health Care.

