# Supplementary material for: No evidence of a clinically important effect of adding local infusion analgesia administrated through a catheter in pain treatment after total hip arthroplasty: A randomized double-blind and placebo-controlled clinical trial involving 60 patients
Source: Acta Orthop. 2011 Jul 8;82(3):315–20. doi: 10.3109/17453674.2011.570671 (PMC3235309; doi:10.3109/17453674.2011.570671)
Supplement: Supplementary file 1 [file ORT-1745-3674-82-315-s4132.pdf]

Supplementary article data

No evidence of a clinically important effect of adding local infusion analgesia administrated through a catheter in pain treatment after total hip arthroplasty

A randomized double-blind and placebo-controlled clinical trial involving 60 patients

Kirsten Specht[1], Jane Schwartz Leonhardt[1], Peter Revald[1], Hans Mandøe[2], Else Bay Andresen[2], John Brodersen[3], Svend Kreiner[4], and Per Kjaersgaard-Andersen[1]

[1]Clinical Research Unit, Department of Orthopaedics, and [2]Department of Anesthesiology, Vejle Hospital; [3]Department and Research Unit of General Practice and [4]Department of Biostatistics, Institute of Public Health, University of Copenhagen, Denmark
Correspondence: kirsten.specht@slb.regionsyddanmark.dk
Submitted 10-04-24. Accepted 10-12-13

Table 1. Item-fit statistics comparing the observed and expected correlations between items and associated rest scores

	Expected	Observed	p-value
Appetite	0.943	0.940	0.8
Nausea	0.949	0.951	0.9
Vomiting	0.792	0.795	1.0

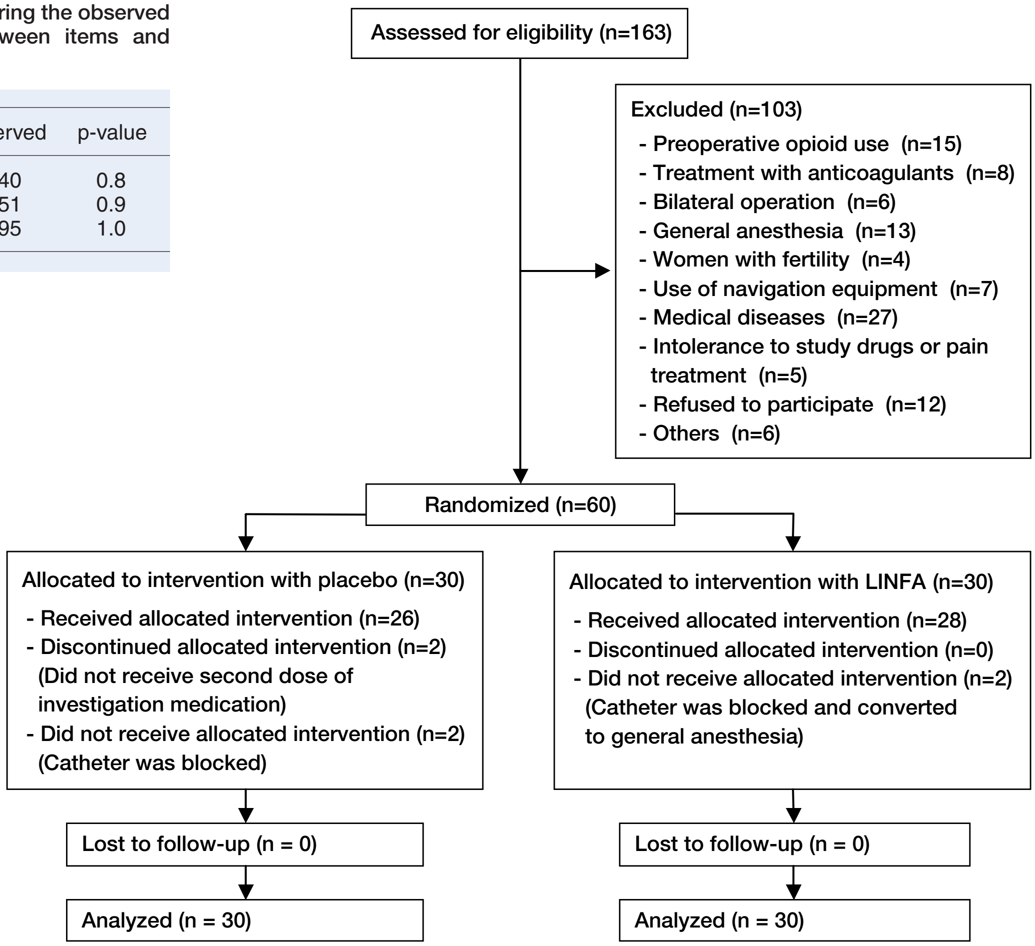

Flow chart of the patients in the study.

Open Access - This article is distributed under the terms of the Creative Commons Attribution Noncommercial License which permits any noncommercial use, distribution, and reproduction in any medium, provided the source is credited.
DOI 10.3109/17453674.2011.570671

Table 3. Consumption of opioids (in mg) in the LINFA and placebo groups, median (range)

	LINFA group (n = 30)	Placebo group (n = 30)	p-value [a]
0–24 hours [b]	27 (0–101)	33 (0–118)	0.5
24–48 hours	7 (0–53)	13 (0–67)	0.2
48–72 hours	7 (0–40)	7 (0–47)	0.7
Day 7	0 (0–27)	0 (0–20)	0.8

[a] Mann-Whitney U test.
[b] Primary endpoint.

Table 5. Results for postoperative pain as secondary endpoint, median (range)

Pain scale	LINFA group	Placebo group	n (L/P) [a]	p-value [b]
Day 1 at 8 p.m.				
WOMAC	4.5 (0–9)	5 (0–9)	30/30	0.6
NRS [c]	4.5 (0–12)	6 (0–15)	30/29	0.3
WOMAC + NRS [c]	9.5 (0–20)	10 (0–24)	30/29	0.3
Day 2 at 8 a.m.				
WOMAC	5 (0–15)	5 (2–12)	29/30	1.0
NRS [c]	4 (0–14)	4.5 (1–15)	29/30	0.7
WOMAC + NRS [c]	9 (0–25)	9.5 (4–27)	29/30	0.8
Day 2 at 8 p.m.				
WOMAC	4 (0–8)	4 (0–12)	30/30	0.3
NRS [c]	4 (0–10)	3 (0–14)	30/30	0.9
WOMAC + NRS [c]	8 (0–18)	6.5 (0–26)	30/30	0.6
Day 3 at 8 a.m.				
WOMAC	7 (0–16)	6 (0–15)	22/21	0.3
NRS [c]	3 (0–10)	3 (0–12)	30/29	0.8
WOMAC + NRS [c]	10 (0–22)	10 (0–24)	22/20	0.4
Day 3 at 8 p.m.				
WOMAC	5 (0–12)	5 (0–10)	27/21	0.4
NRS [c]	3.5 (0–10)	3 (0–15)	28/29	0.9
WOMAC + NRS [c]	9 (0–22)	8 (0–18)	27/21	0.4
Day 7				
WOMAC	7 (2–16)	5.5 (0–11)	25/22	0.2
NRS [c]	3 (0–12)	2 (0–15)	30/30	0.5
WOMAC + NRS [c]	10 (3–28)	10 (0–20)	25/22	0.3

[a] n: number of registrations in the two groups (LINFA/placebo).
[b] Mann-Whitney U test.
[c] NRS pain (rest + activity)
